# *Trapa bispinosa* Roxb. and lutein ameliorate cataract in type 1 diabetic rats

**DOI:** 10.3164/jcbn.19-34

**Published:** 2019-11-12

**Authors:** Sho Kinoshita, Hikari Sugawa, Tomoaki Nanri, Rei-ichi Ohno, Jun-ichi Shirakawa, Hikari Sato, Nana Katsuta, Shiori Sakake, Ryoji Nagai

**Affiliations:** 1 Graduate School of Agriculture, Tokai University, Toroku 9-1-1, Higashi-ku, Kumamoto 862-8652, Japan; 2 Santen Pharmaceutical Co., Ltd., Nihonbashi Muromachi 1-13-7, Chuo-ku, Tokyo 103-0022, Japan; 3 Department of Bioscience School of Agriculture, Tokai University, Toroku 9-1-1, Higashi-ku, Kumamoto 862-8652, Japan

**Keywords:** advanced glycation end products, oxidation, *Trapa bispinosa* Roxb., diabetes mellitus, cataract

## Abstract

*Trapa bispinosa* Roxb. is an annual aquatic grass of the citrus family. Although its hot water extract displays antioxidative activity *in vitro*, little is known about its biological effectiveness. In the present study, we evaluated the extract’s inhibitory effect on diabetic cataractogenesis and formation of advanced glycation end-product. Lutein, which is beneficial for eye diseases, was administered concurrently. For short-term administration, *Trapa bispinosa* Roxb. hot water extract and/or lutein were administered to type 1 diabetic rats. *N*^ɛ^-(carboxymethyl)lysine and *N*^ɛ^-(carboxyethyl)lysine were quantified in serum using mass spectrometry. The long-term administration study was similar to the short-term, except that the dosages were lower. In the short-term study, co-administration of the extract and lutein inhibited *N*^ɛ^-(carboxymethyl)lysine and *N*^ɛ^-(carboxyethyl)lysine in serum. However, in the long-term study, only lutein inhibited *N*^ɛ^-(carboxymethyl)lysine and *N*^ɛ^-(carboxyethyl)lysine in serum. These results suggest that lutein exerts its long-term effect regardless of the concentration administered, while the extract exerts its effect when its concentration is increased. Relative to the consumption of the control diet, oral intake of the combination of the extract and lutein significantly inhibited the progression of cataractogenesis in the lens of diabetic rats, even at low doses, and the combination was more effective than individual treatments.

## Introduction

The crystalline lens is normally a water soluble and clear protein. However, when aggregated it becomes insoluble and opaque, which decreases the permeability of light reaching the retina, resulting in the loss of visual acuity.^(1)^ Clinical evidence has shown that aging and diabetes might be involved in the development of cataracts.^(2,3)^ According to a 2010 report by the World Health Organization, the prevalence of cataracts has been increasing each year, accounting for approximately 51% of blind cases worldwide. Approximately 20 million patients are believed to suffer from this disorder.^(4)^ The onset of cataracts is caused by several factors, such as oxidation,^(5)^ deamidation,^(6)^ nonenzymatic glycation,^(7,8)^ and isomerization^(9)^ of amino acids constituting lens proteins. To date, however, there is no effective chemotherapy for cataracts. Instead, a lens affected by cataracts is typically surgically replaced with an artificial lens. Hence, preventing the onset of the cataracts is important.

Lutein is a type of carotenoid that exists in the crystalline lens and the macular region of the eye. It is an antioxidant that is not synthesized *in vivo*.^(10)^ Previous studies have reported a reduction in the risk of age-related macular degeneration and the development of cataracts.^(11–13)^

*Trapa bispinosa* Roxb. (*T. bispinosa* Roxb.) is an annual aquatic grass of the citrus family that is widely used in Asia and globally as an edible and medicinal plant. Plants from the *Trapa* genus have been reported to have various physiological functions including antioxidant,^(14)^ antimicrobial,^(15)^ and antiulcer activity.^(16)^ In addition, recent studies have demonstrated that the *Trapa *genus inhibits the formation of cross-linking^(17)^ and carbonylation of α-crystallin^(18)^ by glycation *in vitro*. However, little is known about the biological function of *T. bispinosa* Roxb.

The nonenzymatic Maillard reaction between proteins and reducing sugars progresses in a variety of proteins *in vivo*, such as hemoglobin, albumin, and collagens. This is followed by the formation of advanced glycation end products (AGEs). Recent studies have demonstrated that AGE accumulation *in vivo* is involved in aging and lifestyle-related diseases, such as diabetes and atherosclerosis.^(19)^ For instance, the level of *N*^ɛ^-(carboxymethyl)lysine (CML) and *N*^ɛ^-(carboxyethyl)lysine (CEL) is significantly increased by diabetes^(20,21)^ with the typical complication being cataracts in both rats and humans.^(22)^ In this study, we measured the inhibitory effects of lutein, which displays antioxidant activity, and *T. bispinosa* Roxb. hot water extract (TBE) on the formation of AGEs and cataractogenesis in diabetic rats.

## Materials and Methods

### Chemicals

Gallic acid and ellagic acid were purchased from Tokyo Chemical Industry Co., Ltd. (Tokyo, Japan). Eugeniin was purchased from Nagara Science Co., Ltd. (Gifu, Japan). Lutein was purchased from Koyo Mercantile Co., Ltd. (Tokyo, Japan). TBE (67% *T. bispinosa* Roxb. peel extract) was purchased from Hayashikane Sangyo Co., Ltd. (Yamaguchi, Japan). Isotonic sodium chloride solution was purchased from Otsuka Pharmaceutical Factory (Tokushima, Japan). The blood glucose level measurement kit was purchased from ARKRAY, Inc. (Kyoto, Japan). Isoflurane was purchased from Mylan Inc. (Tokyo, Japan). All other chemicals were of the best grade available from commercial sources.

### Measurement of total polyphenol content in TBE

 Total polyphenol was measured using the Folin-Ciocalteu method as described previously.^(23,24)^ Briefly, TBE (100–200 mg) in 50% ethanol was solubilized using a sonicator. After addition of the Folin-Ciocalteu reagent to the filtrate, the reaction mixture was analyzed by absorbance at 660 nm. Catechin was used as the standard sample to construct the calibration curve.

### Detection of polyphenols by high-performance liquid chromatography (HPLC)

TBE (60 mg) in 5 ml of 20% acetonitrile was solubilized using a sonicator and filtrates were analyzed by HPLC using a model LC20A apparatus (Shimadzu Corporation, Kyoto, Japan) equipped with an X Bridge column (2.1 mm diameter × 150 mm, 5 µm; Nihon Waters, Tokyo, Japan). In the gradient analysis, the mobile phase A was 0.1% formic acid and mobile phase B was 0.1% formic acid acetonitrile. The mobile phase B was 5% from 0 to 10 min, 10% from 10 to 20 min, 15% from 20 to 30 min, and 50% from 30 to 40 min at a flow rate of 0.3 ml/min. The column temperature was set at 40°C and 5 µl of the sample was injected. The absorbance at 280 nm was monitored. The eluted substance was characterized based on retention time.

### Animal experiments

All animal experiments were confirmed by Ina Research Inc. (Nagano, Japan; approval number for short-term administration: 17192; approval number for long-term administration: 16034). Experiments were conducted in compliance with the Guidelines for the Care and Use of Animals for scientific purposes at Ina Research Inc., established on April 22, 2014. Wistar rats were purchased from Charles River Laboratories Japan, Inc. (Kanagawa, Japan). Rats were housed in a pathogen-free barrier facility (12 h light–dark cycle) and fed a normal rodent chow diet (Oriental Yeast Co., Ltd., Tokyo, Japan).

### Short-term administration

Diabetes mellitus (DM) was induced in 5-week-old male rats by a single intravenous (tail vein) injection of streptozotocin (60 mg/kg body weight) in an isotonic sodium chloride solution. One week after diabetes induction (blood glucose ≥200 mg/dl), rats were randomly divided into one diabetic untreated group (*n* = 8) and three diabetic treated groups; the latter groups received TBE 100 mg/kg body weight/day (DM-TBE100; *n* = 8), lutein 10 mg/kg body weight/day (*n* = 8), or TBE 100 mg/kg body weight/day and lutein 10 mg/kg body weight/day (*n* = 8). All groups including the normal group (non-diabetic group; *n* = 8) were fed a normal rodent chow diet. TBE and lutein were administered orally every day via a blunt-tipped feeding needle for 29 days. Body weight and blood glucose level were measured every week in each rat. Thirty days after diabetes induction, animals were euthanized under isoflurane anesthesia and serum was extracted.

### Long-term administration

Animal experiments were performed according to the methods described for the short-term administration. Rats were randomly divided into one diabetic untreated group (*n* = 8) and three diabetic treated groups; the latter groups received TBE 2 mg/kg body weight/day (DM-TBE2; *n* = 8), lutein 0.4 mg/kg body weight/day (*n* = 8), or TBE 2 mg/kg body weight/day and lutein 0.4 mg/kg body weight/day (DM-TBE2 + Lut0.4; *n* = 8). All groups including the normal group (non-diabetic group; *n* = 8) were fed a normal rodent chow diet. TBE and lutein were administered orally every day via a blunt-tipped feeding needle for 69 days. Body weight was measured every week and the blood glucose level was measured every month in each rat. Seventy days after diabetes induction, animals were euthanized under isoflurane anesthesia and serum and the lenses were extracted from rats.

### Measurement of CML and CEL in serum by LC-MS/MS

CEL and CML contents in rat serum were measured by electrospray ionization liquid chromatography-tandem mass spectrometry (ESI-LC-MS/MS) using a TSQ Quantiva triple-stage quadrupole mass spectrometer (Thermo Fisher Scientific, Waltham, MA) as described previously.^(25)^ In brief, 5 µl of serum and 15 µl of distilled water were mixed with 20 µl of 200 mM sodium borate buffer (pH 9.1) and reduced by the addition of 2 µl of NaBH_4_ (1 M NaBH_4_ in 0.1 N NaOH) at 25°C for 4 h. Standard 10 pmol of [^2^H_4_] CEL, [^2^H_2_] CML (PolyPeptide Laboratories, Strasbourg, France), and 5 nmol of [^13^C_6_] lysine (Cambridge Isotope Laboratories, Inc., Tewksbury, MA) were added to the pellets, and samples were hydrolyzed with 1 ml of 6 N HCl at 100°C for 18 h. The samples were dried *in vacuo*, resuspended in distilled water, then passed over a Strata-X-C column (Phenomenex, Torrance, CA), which was pre-washed with 1 ml of methanol and equilibrated with 1 ml of distilled water. The column was then washed with 3 ml of 2% formic acid and eluted with 3 ml of 7% ammonia. The pooled elution fractions were dried and resuspended in 1 ml of 20% acetonitrile containing 0.1% formic acid. Ten microliters was used for ESI-LC-MS/MS. LC was carried out using a ZIC^®^-HILIC column (150 × 2.1 mm, 5 µm; Merck Millipore, Billerica, MA). The mobile phase consisted of solvent A (distilled water containing 0.1% formic acid) and solvent B (acetonitrile containing 0.1% formic acid). The flow rate was 0.2 ml/min, and the column temperature was maintained at 40°C. The retention times for CEL, CML, and lysine were 12–15 min. CEL, CML, lysine, and the standard were detected by electrospray-positive ionization-MS multiple reaction monitoring. The parent ions of CEL and [^2^H_4_] CEL were 219 (*m*/*z*) and 223 (*m*/*z*), respectively. Fragment ions of 130 (*m*/*z*) and 134 (*m*/*z*) from each parent ion were measured for the analysis of CEL and [^2^H_4_] CEL in samples. The parent ions of CML and [^2^H_2_] CML were 205 (*m*/*z*) and 207 (*m*/*z*), respectively. The parent ions of lysine and [^13^C_6_] lysine were 147 (*m*/*z*) and 153 (*m*/*z*), respectively. Fragment ions of 84 (*m*/*z*) and 89 (*m*/*z*) from each parent ion were measured for the analysis of lysine and [^13^C_6_] lysine in samples. Fragment ions of 130 (*m*/*z*) from each parent ion were measured for the analysis of CML and [^2^H_2_] CML in samples. CEL and CML were normalized to the lysine content.

### Measurement of opacity level of crystalline lens

 Cataracts were graded on a 1 to 4 scale by placing the lenses on a grid sheet, as described previously.^(26,27)^

### Statistical analyses

Data with homogeneity (body weight, and CML and CEL contents) were analyzed using one-way analysis of variance (ANOVA). Multiple post-hoc comparisons were performed using Dunnett’s test.^(28)^ Data without homogeneity (blood glucose levels and cataract grade) and multiple post-hoc comparisons were performed with Steel’s test.^(29)^

## Results

### Polyphenol content and composition in TBE

**Fig. 1:**
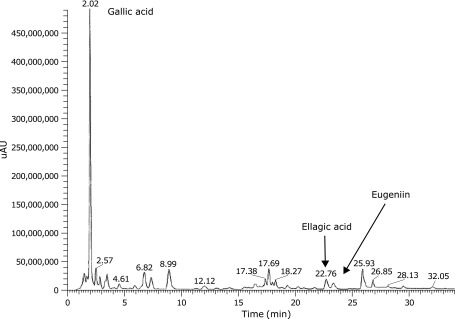
Measurement of polyphenol in TBE by HPLC. A 5 µl aliquot of TBE (12 mg/ml) was injected in the HPLC apparatus equipped with an X Bridge column and absorbance at 280 nm was monitored. The eluted substance was characterized based on retention time.

The amount of polyphenol in TBE was estimated to be 25% w/w. HPLC demonstrated the presence of multiple polyphenols including gallic acid, ellagic acid, and eugeniin in TBE (Fig. 1).

### Inhibitory effect of TBE and lutein on CML and CEL accumulation *in vivo* (short-term administration)

**Fig. 2:**
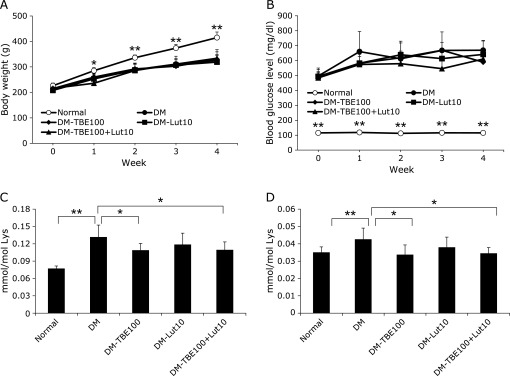
Biochemical parameters and quantification of AGEs in rats for short-term administration. Changes in body weight and blood glucose level of rats. Diabetes was induced in rats with streptozotocin, and changes in body weight (A) and blood glucose levels (B) were measured. The level of CML (C) and CEL (D) in rat serum was measured by LC-MS/MS, as described in Materials and Methods. Normal: normal group (open circle, *n* = 8), DM: diabetes mellitus group (closed circle, *n* = 8), DM-TBE100: diabetes mellitus group treated with 100 mg/kg body weight/day TBE (closed diamond, *n* = 7), DM-Lut10: diabetes mellitus group treated with 10 mg/kg body weight/day lutein (closed square, *n* = 8), DM-TBE100 + Lut10: diabetes mellitus group treated with 100 mg/kg body weight/day TBE and 10 mg/kg body weight/day lutein (closed triangle, *n* = 8). Data are presented as mean ± SD. *p<0.05, **p<0.01 vs Diabetes mellitus group (DM).

To evaluate the progression of diabetes, body weight (Fig. 2A) and the level of fasting blood glucose (Fig. 2B) were measured every week. The body weight of normal rats increased from 200 to 400 g, while that of diabetic rats increased to 330 g during the 29-day experimental period (Fig. 2A). This increase in body weight of normal rats was significant and time-dependent, whereas that of diabetic groups was gradual. The blood glucose level of normal rats was maintained below 120 mg/dl, while that of diabetic rats increased from 500 to 670 mg/dl during the 29-day experimental period (Fig. 2B). The level of blood glucose between the normal and diabetic group was statistically significant from the beginning of the experimental period. Compared to the diabetes mellitus group, there was no significant change in body weight and glucose levels in the TBE and lutein administration group. During the experimental period, one of the DM-TBE100 group members died. For the measurement of the inhibitory effects of TBE and lutein on the formation of CML and CEL *in vivo*, TBE 100 mg/kg body weight, lutein 10 mg/kg body weight, and TBE 100 mg/kg body weight with lutein 10 mg/kg body weight were administered orally every day via a blunt-tipped feeding needle to diabetic rats for 29 days. The CML and CEL in the serum of the diabetic group were significantly increased compared to those in the normal group (Fig. 2C and D) (*p*<0.01). The accumulation of CML and CEL was significantly inhibited when TBE and TBE with lutein were administered (*p*<0.05).

### Effect of TBE and lutein on CML and CEL accumulation *in vivo* (long-term administration)

**Fig. 3:**
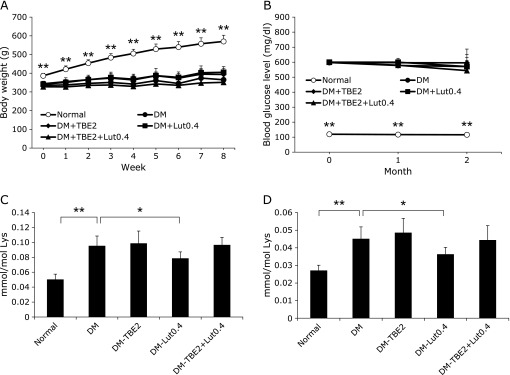
Biochemical parameters and quantification of AGEs in rats for long-term administration. Changes in body weight and blood glucose level of rats. Diabetes was induced in rats with streptozotocin, and changes in body weight (A) and blood glucose levels (B) were measured. The level of CML (C) and CEL (D) in the serum of rats was measured by LC-MS/MS, as described in Materials and Methods. Normal: normal group (open circle, *n* = 8), DM: diabetes mellitus group (closed circle, *n* = 8), DM-TBE2: diabetes mellitus group treated with 2 mg/kg body weight/day TBE (closed diamond, *n* = 6), DM-Lut0.4: diabetes mellitus group treated with 0.4 mg/kg body weight/day lutein (closed square, *n* = 8), and DM-TBE2 + Lut0.4: diabetes mellitus group treated with 2 mg/kg body weight/day TBE and 0.4 mg/kg body weight/day lutein (closed triangle, *n* = 6). Data are presented as mean ± SD. *p<0.05, **p<0.01 vs Diabetes mellitus group (DM).

As the administration of TBE and TBE with lutein for 1-month suppressed the level of CML and CEL in serum, these compounds were administered at a low dosage for a longer period to evaluate their biological significance. Over 69 days, the body weight of normal rats increased from 350 to 570 g, while that of diabetic rats increased to 360 g (Fig. 3A). Similar to short-term administration study, this increase in body weight of normal rats was significant and time-dependent, whereas that of diabetic groups was gradual. The blood glucose level of normal rats remained below 120 mg/dl throughout the experimental period, while that of diabetic rats was maintained at 600 mg/dl (Fig. 3B). The level of blood glucose between the normal and diabetic groups was statistically significant from the beginning of the experimental period. There was no significant change in body weight and glucose levels when TBE and lutein were administered to the DM group. During the experimental period, two rats in the DM-TBE2 group and two in the DM-TBE2 + Lut0.4 group died. As previously mentioned, TBE, lutein, and TBE with lutein were administered orally every day via a blunt-tipped feeding needle to diabetic rats for 69 days. The level of CML and CEL in the serum was increased by approximately 2-fold under diabetic conditions (Fig. 3C and D) (*p*<0.01). Furthermore, treatment with lutein significantly reduced the formation of CML and CEL (*p*<0.05), whereas TBE and TBE with lutein did not show an inhibitory effect.

Before performing these experiments, we measured the LD_50_ of TBE in mice. Although 2,000 and 4,000 mg/kg body weight of TBE were orally administered to 7-week-old male ddY mice (*n* = 6 per group), there was no detectable TBE toxicity in the liver, kidney, spleen, lung, and heart after 24 h. Thus, the LD_50_ of TBE was more than 4,000 mg/kg body weight (data not shown). In the present study, 1/2,000 of the mouse LD_50_ of TBE was administered to rats. We concluded that a few diabetic rats died from causes other than TBE since there were no detectable side effects, such as alterations in body weight and blood glucose levels (Fig. 2A and B, 3A and B).

### Inhibitory effect of TBE and lutein on cataractogenesis

**Fig. 4:**
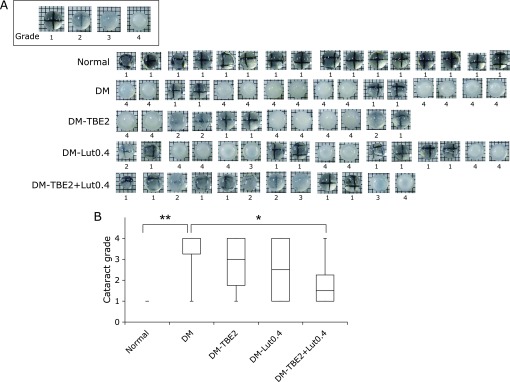
Evaluation of cataract levels. Rat lenses were removed from the eyes and backscattering of light was determined by placing the lenses on a grid sheet with a 1 mm spacing (A). Criteria for grading: 1, clear lens (grid lines were clearly visible). 2, lens with a slightly cloudy appearance (grid lines were visible). 3, lens with a cloudy appearance (part of grid lines can be confirmed). 4, lens was completely cloudy (grid lines could not be seen). Cataract progression was compared in each group (B). Normal: normal group (*n* = 16), DM: diabetes mellitus group (*n* = 16), DM-TBE2: diabetes mellitus group treated with 2 mg/kg body weight/day TBE (*n* = 12), DM-Lut0.4: diabetes mellitus group treated with 0.4 mg/kg body weight/day lutein (*n* = 16), and DM-TBE2 + Lut0.4: diabetes mellitus group treated with 2 mg/kg body weight/day TBE and 0.4 mg/kg body weight/day lutein (*n* = 12). Data are presented as median ± max and minimum. *p<0.05, **p<0.01 vs Diabetes mellitus group (DM).

Lenses were examined under a microscope and photographed. The degree of cataract formation was quantified by visual inspection and grading.^(26,27,30)^ As shown in Fig. 4A, many diabetic rats developed severe cataracts. Administering TBE and lutein to diabetic rats lessened cataractogenesis, with the combination treatment producing a significant amelioration.

## Discussion

Glycation affects the denaturation and inactivation of proteins *in vivo*, and this is followed by conformational changes of proteins and a decrease in enzymatic activity.^(31)^ The binding of AGEs to their receptor has been reported to induce the expression of inflammatory cytokines, thereby contributing to the progress of age-related diseases.^(32)^ TBE inhibits glycation of α-crystallin *in vitro*.^(18)^ Furthermore, lutein exhibits antioxidative activity and protects the crystalline lens from cataractogenesis.^(10,13)^ Since the formation of CML, a major antigenic AGE structure,^(33)^ requires glycation and oxidation, we expected the use of these two compounds would more effectively ameliorate cataractogenesis. In the present study, CML and CEL in rat serum were significantly increased by the induction of diabetes (Fig. 2C and D). Also, the basal AGE levels in the serum of normal rats were different between short-term and long-term studies, even using the same strains, likely because body weights during the feeding period were also changed. The dosages of TBE and lutein were decided based on previous studies with natural compounds. Kong *et al.*^(34)^ orally administered berberine isolated from a Chinese herb to hamsters at doses of 50 and 100 mg/kg body weight/day to investigate its effects. We previously administered purified esculeoside A isolated from tomato to apoE-deficient mice at doses of 50 and 100 mg/kg body weight/day.^(35)^ Therefore, for the short-term administration, 100 mg/kg body weight of TBE was used, whereas 10 mg/kg body weight of lutein was used since this dosage is already 50-fold higher than the dose used in a human clinical study.^(11,12)^ With respect to long-term administration, since TBE at 100 mg/kg body weight/day had an inhibitory effect in a short-term study, we next measured its effects at a lower dosage. For lutein, 0.4 mg/kg body weight/day was used, which was the same dosage as that used in a human clinical trial.^(36)^ Although short-term administration of TBE significantly suppressed the level of serum CML and CEL in diabetic rats, it did not inhibit serum AGE levels when administered long-term. The concentration of TBE used for the long-term administration was 2 mg/kg body weight, a low concentration when converted to human dosages. This indicates that TBE may inhibit AGEs *in vivo* by increasing the amount administered. Furthermore, lutein did not suppress AGEs when administered short-term (Fig. 2C and D). However, suppression was observed during long-term administration (Fig. 3C and D).

It has been reported that a rise in lutein concentration in blood does not occur immediately after administration, but instead occurs approximately 10 weeks after administration.^(37)^ Therefore, administering a high lutein concentration for 29 days might be insufficient to suppress AGE in the blood. CML is one of the major AGE structures generated by an oxidation reaction.^(38)^ In contrast, CEL is generated from both methylglyoxal (MG) and acetol, a metabolite formed from acetone by acetone monooxygenase. Oxidation is required when protein reacts with acetol to form CEL.^(26)^ However, we still do not know what percentage of CEL *in vivo* is generated from acetol. TBE exhibits scavenging activity, with an IC_50_ of 492 ± 0.7 µg/ml and 7 ± 0.76 µg/ml for 2,2-diphenyl-1-picrylhydrazyl and 2,2'-azino-bis(3-ethylbenzothiazoline-6-sulphonic acid) radicals, respectively.^(39)^ Taken together, the antioxidative activity of TBE and lutein may suppress the formation of CML, and CEL is suppressed by preventing the aggravation of diabetes by natural product administration *in vivo*.

When cataracts were evaluated, we identified that there was a tendency to suppress the onset of cataracts when TBE and lutein were administered long-term, and significant amelioration was evident in the cataracts when both TBE and lutein were used. The relationship between the progression of cataracts and AGEs has been examined in several studies.^(40,41)^ However, we did not observe a suppression of AGE accumulation in the lenses by administering TBE and lutein (data not shown), suggesting that CML and CEL were not directly related to the pathogenesis of cataracts or other AGEs are involved. As the clearance of lens proteins is slow, it is considered that a significant difference does not exist when natural products are administered. Nevertheless, to our knowledge, this study provides the first evidence that TBE and lutein inhibit cataractogenesis even at a practical concentration and are more effective when ingested together. Since the average body weight of diabetic rats is about 370 g (Fig. 2A), the dose of lutein (0.4 mg/kg body weight/day) was 0.15 mg/rat/day. This dosage would be 20 mg/day for an average 50 kg human. Likewise, the dose of TBE (2 mg/kg body weight/day) would be 100 mg/day. Lutein, at dosages 100–200-fold higher than used in the present study, have been used in an animal study.^(42)^ While TBE has not yet been tested animally, a crude extract of natural compounds such as *Satureja hortensis* L. would likely be administered at 500–2,000 mg/kg body weight/day, 250–1,000-fold higher than the present study.^(43)^ Thus, concerning the dose of long-term experiment, both the crude extract of TBE at a dosage of 2 mg/kg body weight and lutein with a dosage of 0.4 mg/kg body weight are more practical in concentration than the dosages of natural products often reported.

## Author Contributions

SK, HS, RO, HS, NK, and SS, acquisition of data; TN and RN, study concept and design; JS, analysis and interpretation of data.

## Abbreviations

AGEs: advanced glycation end products
CEL: *N*^ɛ^-(carboxyethyl)lysine
CML: *N*^ɛ^-(carboxymethyl)lysine
DM: diabetes mellitus
ESI-LC-MS: electrospray ionization liquid chromatography-tandem mass spectrometry
HPLC: high-performance liquid chromatography
MG: methylglyoxal
TBE: *Trapa bispinosa* hot water extract
